# Increased Nitric Oxide and Attenuated Diastolic Blood Pressure Variability in African Americans with Mildly Impaired Renal Function

**DOI:** 10.4061/2010/137206

**Published:** 2011-01-09

**Authors:** Keith M. Diaz, Deborah L. Feairheller, Kathleen M. Sturgeon, Praveen Veerabhadrappa, Sheara T. Williamson, Deborah L. Crabbe, Michael D. Brown

**Affiliations:** ^1^Hypertension, Molecular and Applied Physiology Laboratory, Department of Kinesiology, College of Health Professions, Temple University, Philadelphia, PA 19122, USA; ^2^Division of Cardiology, Department of Medicine, Temple University Health Sciences Campus, Philadelphia, PA 19122, USA; ^3^Cardiovascular Research Center, School of Medicine, Temple University, Philadelphia, PA 19122, USA

## Abstract

We investigated the relationship between renal function, blood pressure variability (BPV), and nitric oxide (NO) in a group of African Americans with normal or mildly impaired renal function. 24-hour ambulatory blood pressure monitoring was performed, NO measured, and glomerular filtration rate (GFR) calculated in 38 African Americans. Participants were categorized as having normal (GFR > 90 mL/min per 1.73 m^2^) or mildly impaired (GFR 60–89 mL/min per 1.73 m^2^) renal function. Diastolic BPV was significantly lower in the mildly impaired renal function group. Regression analyses revealed a significant positive association between GFR and diastolic BPV for the entire study group. Plasma NO levels were significantly higher in the mildly impaired renal function group and negatively correlated with diastolic BPV. In conclusion, diastolic BPV was reduced in African Americans with mildly impaired renal function, which may be the result of increased NO production. These results conflict with previous findings in diseased and nonblack populations and could provide rationale for studying BPV early in the disease state when BP-buffering mechanisms are still preserved.

## 1. Introduction

Blood pressure (BP) levels are the product of both environmental stimuli and internal regulatory mechanisms of the cardiovascular and renal systems. As a result of the complex interaction between extrinsic and intrinsic factors, BP undergoes continuous fluctuations over a 24-hour period. With the development of noninvasive ambulatory BP monitoring, blood pressure variability (BPV), a surrogate marker for the complex interaction between external and internal factors, can be quantified to assess the clinical importance of daily fluctuations in BP. Cross-sectional studies have reported that daytime and 24-hour BPV are both directly associated with the prevalence and severity of target-organ damage (TOD), independent of mean BP [1–4]. These cross-sectional data have been confirmed by longitudinal studies, as BPV has been identified as an independent predictor of cardiovascular mortality [5, 6]. The association between BPV and cardiovascular risk may be attributed to the augmented mechanical stress placed on the vasculature that occurs as a result of increased variability of blood flow. The augmented mechanical stress may in turn facilitate vascular remodeling and contribute to the advancement in atherosclerosis, thereby conferring greater cardiovascular risk. 

In the renal vasculature, the augmented mechanical stress characterized by greater BPV may lead to injury, activation, and dysfunction of the glomerular endothelium. Therefore, BPV may be a contributing factor in the progression of renal disease by exerting a detrimental effect on the glomerular endothelium. Cross-sectional studies support a role for BPV in renal dysfunction, as a direct association between BPV and renal damage, as indicated by microalbuminaria, has been found [4, 7, 8]. Recently, it has been reported that the rate of BPV, which accounts for both the magnitude of BPV and the time elapsed between two successive BP readings in quantifying how fast or slow BP values change, was a significant predictor of impaired renal function in a large cohort of adults with essential hypertension, suggestive that the rate of BPV may also have clinical importance for renal function [9]. Taken together, these results suggest that both the magnitude and rate of BPV may contribute to the accelerated progression of renal disease. However, the association between BPV and renal function has not been previously investigated in the early stages of renal disease when BP-buffering mechanisms, including baroreceptor and endothelial function, are still preserved. To better elucidate this relationship and implicate BPV in the acceleration of renal disease, studies in the early stages of renal disease are needed.

The purpose of this study was to investigate the relationship between BPV and renal function in a group of normotensive and pre-hypertensive African Americans with normal or mildly impaired renal function. Because the release of nitric oxide (NO) from the endothelium may serve as a BP-buffering mechanism in response to fluctuations in blood flow and shear stress [10], a secondary purpose of this study was to assess the potential association between NO and BPV in this cohort of African Americans.

## 2. Methods

### 2.1. Participants

Participants were recruited via mailed brochures and local newspaper advertisements. Upon response to either, the participants were contacted by telephone to assess their eligibility. Each participant gave written informed consent following the explanation of study protocols during their first laboratory visit. The protocol was approved by the Temple University Institutional Review Board. 

African American men and women between the ages of 40 to 75 years of age who were sedentary (regular aerobic exercise <2 day per week), nonsmoking, nonmorbidly obese (BMI < 40 kg/m^2^), not on lipid-lowering medication, and had no history of cardiovascular disease, diabetes, hypercholestermia, liver disease, renal disease, or lung disease were initially enrolled in the study. Both pre-menopausal and post-menopausal women were included in the study; all post-menopausal women were not on hormone replacement therapy. Participants on more than one anti-hypertensive medication were excluded from the study.

### 2.2. Screening

To ensure the eligibility of all qualified participants, three screening visits were completed prior to inclusion in the study. Screening visit one consisted of blood sampling and urinalysis following a 12-hour overnight fast to assess blood chemistries and renal function. Any individual who had total cholesterol >240 mg/dL or who had fasting blood glucose >126 mg/dL were excluded from the study. Glomerular Filtration Rate (GFR) was calculated using the four-variable Modification of Diet in Renal Disease (MDRD) study equation specific to African Americans [11]: GFR (mL/min per 1.73 m^2^) = 186 × (SCr)^−1.154^ × (Age)^−0.203^ × (0.742 if female) × (1.21 if black), where SCr represents serum creatinine. Any individual who exhibited evidence of renal disease (GFR < 60 mL/min per 1.73 m^2^) was excluded from the study. Participants were categorized as having normal (GFR > 90 mL/min per 1.73 m^2^) or mildly impaired GFR (GFR 60–89 mL/min per 1.73 m^2^) according to National Kidney Foundation criteria.

Screening visits two and three required all qualified participants to undergo a physician administered physical examination and echocardiogram bicycle stress test to confirm that participants displayed no evidence of cardiovascular, pulmonary, or other chronic diseases.

### 2.3. Dietary Stabilization

Participants who met all inclusion criteria after screening underwent dietary stabilization for 6 weeks prior to testing. Any participant receiving anti-hypertensive monotherapy (*n* = 14) was tapered off of their medication during this dietary stabilization period. Participants were instructed by a Registered Dietician on the American Heart Association (AHA) Dietary Guidelines for Healthy American Adults, a diet formerly known as the AHA step 1 diet. This diet consisted of ~55% of total daily calories from carbohydrates, 15% from protein, and <30% from fat, with saturated fat ≤10% of total calories, sodium ≤3-4 g/day, and cholesterol intake <300 mg/day. Participants met with the dietician one time per week at which time body weight and BP were recorded for each visit. Participants were required to remain within 5% of their study entry body weight for the duration of the study. In addition, participants who exhibited BP consistently greater than 159/99 mmHg during the stabilization period were excluded from the study. Compliance to the prescribed diet was monitored by completion of a 3-day food record at the conclusion of dietary stabilization. All participants who were in compliance with the diet underwent testing 1-2 weeks after dietary stabilization.

### 2.4. Office BP Measurements

Office BP measurements were made in accordance with JNC 7 guidelines on three separate occasions. BP was measured using a mercury sphygmomanometer after 5 minutes of quite rest in a chair, with feet on the floor and arm supported at heart level. An appropriate-size cuff was applied around the participant's nondominant arm and BP values were identified from the first and fifth phase of Korotokoff sounds. BP measurements were performed in triplicate, 5 min apart, and the average of the three values was used as the BP for the visit.

### 2.5. 24-Hour Ambulatory BP Monitoring and Urine Collection

Participants underwent 24-hour ambulatory BP monitoring (ABPM) using a non-invasive monitor (SpaceLabs Medical Inc., Model 90219, Redmond, WA) beginning on the morning of each participant's typical day, with the exclusion of Friday through Sunday. The BP cuff was fitted to the participant's nondominant arm with cuff size determined by upper arm circumference. BP measurements were obtained at 30 minute intervals during the day (6:00 AM–10:00 PM) and 60 minute intervals at night (10:00 PM–6:00 AM). Participants were instructed not to exercise prior to or during the 24-hour BP monitoring period and to pause momentarily and maintain their body position during each BP measurement. Throughout the duration of the 24-hour recording period, participants were required to maintain a diary in which they recorded their activity and emotional status at the time of each BP measurement. Only recordings of good technical quality (>80% of valid BP measurements) were included in final analyses. 

24-hour urine collection occurred on the same day as ABPM. Total volume of urine was measured and recorded; thereafter, urine samples were sent to Quest Diagnostics for measurement of urinary creatinine, urinary sodium (Na^+^), and urinary albumin levels.

### 2.6. Analysis of ABPM Data

Awake and sleep periods were defined according to self-reported sleep times recorded in participants' diaries. 24-hour, awake, and sleep mean values were calculated for systolic BP (SBP), diastolic BP (DBP), mean arterial pressure (MAP), and heart rate (HR). Participants were categorized as pre-hypertensive (120/80–139/89) or normotensive (<120/80 mmHg) according to their mean 24-hour BP. Any participants with mean 24-hour BP >140/90 mmHg were excluded from analyses. ABPM measurements were used instead of office BP measurements under the assumption that BP measurements obtained during ABPM reflect a participant's BP in their natural environment, outside the influence of experimental settings. Because current criteria for ABPM do not have a clearly defined pre-hypertensive category, we defined prehypertension according to JNC 7 guidelines (120/80–139/89 mmHg) [12].

The % dip in BP during sleep was calculated as: (Awake BP − Sleep BP) × (100/awake BP). BPV was calculated using the average real variability (ARV) index [13]

(1) $$\text{ARV}=\frac{1}{N-1}\overset{N-1}{\underset{K=1}{\sum }}\left|\text{B}{\text{P}}_{k+1}-\text{B}{\text{P}}_{k}\right|,$$

where *N* is the number of valid BP measurements and BP_*k*+1_ and BP_*k*_ represent two successive BP measurements. The rationale for selecting the ARV index for BPV calculations is based on previous studies that have reported the ARV index to be a more reliable representation of time series variability than standard deviation or coefficient of variation [13, 14]. 

The rate of BP variation was calculated according to the formula described by Zakopoulos et al. [15]

(2) $$R=\left|\overset{®}{r}\right|=\frac{\sum_{i=1}^{N-1}\left|r_{i}\right|}{N-1},$$

where *N* is the number of valid BP measurements. *r*_*i*_ is defined as follows:

(3) $$r_{i}=\frac{S_{i+1}-S_{i}}{t_{i+1}-t_{i}},$$

at time index

(4) $$t_{ri}=\frac{t_{i}+t_{i+1}}{2},$$

  *S*_*i*_ and *S*_*i*+1_ represent two successive BP measurements at time indices *t*_*i*_ and *t*_*i*+1_. We selected the rate of BP variation as an additional measure of BPV because it accounts for the order in which the BP measurements are obtained, as well as the time between successive readings. Moreover, this parameter permits the evaluation of how fast or slow and in which direction BP values change. The rationale for using both the ARV index and the rate of BP variation was to allow us to quantify the absolute magnitude of BPV (ARV index), while also considering the magnitude of BPV in relation to the time that elapsed between successive BP readings (rate of BP variation).

### 2.7. Bioelectrical Impedance Analysis (BIA)

Body composition was assessed by whole-body BIA using the single-frequency impedance instrument ImpediMed DF50 (San Diego, CA). BIA was measured at 50 kHz on the right side of the body, with two electrodes placed on each dorsal right hand and dorsal right foot while participants were lying in a supine position with their legs slightly apart and hands resting next to the body palms down. Participants were asked to remove all jewelry and other accessories prior to measurements. All electrode sites were cleaned with an alcohol swab before attachment. Measures were taken after at least 10 min of lying in the supine position to reduce possible errors from acute changes in body fluid distribution. Three measurements were taken, and the mean output values of impedance, phase, resistance, and reactance were used for calculations of total fat mass and total lean body mass according to the manufacturer's standard operating procedures.

### 2.8. Measurement of Plasma and Urinary Nitrates/Nitrites (NO*x*)

Blood samples were collected in the morning following a 12-hour overnight fast. Blood was drawn into *K*_2_ EDTA tubes, centrifuged at 2000 g for 20 minutes at 4°C, and then the plasma was frozen at −80°C until assay. On the day of assay, plasma samples were ultrafiltrated through a 10,000 MWCO Amicon Ultra filter (Millipore) by microcentrifuge at 14,000 g for 30 minutes at 4°C. Urine was aliquoted from the total volume 24-hour urine collection and frozen at −80°C until assay. All urine samples were diluted 1:10 in Reaction Buffer (HEPES based). Levels of NO end-products were measured using a modified Griess assay as previously described [16]. Inter-assay and intra-assay CVs were 7.6% and 10.6% respectively.

### 2.9. Statistical Analysis

Data are expressed as means ± the standard error of the mean (SEM). The distribution of all variables was examined using the Shapiro-Wilk test of normality, and homogeneity of variances was determined using Levene's test. Variables that were found to fail the normality test were log adjusted for any statistical analysis, but true physiological values of any variable are reported throughout the paper. Statistical comparisons were performed between normal and mildly impaired renal function groups using the independent *t*-test. Diastolic BPV and the rate of variation in DBP were also compared between the two groups by ANCOVA after adjusting for age, gender, BMI, plasma glucose, cholesterol values, and mean BP levels obtained from office and ABPM measurements. The relationship of each clinical, renal, and BP variable with GFR and urinary albumin were tested using univariate and multivariate regression analyses. *P* values <.05 were considered statistically significant for all analyses. All statistical analyses were performed using SPSS version 17.0 (SPSS Inc., Chicago, IL).

## 3. Results

### 3.1. Clinical, Renal, and BP Characteristics of Normal and Mildly Impaired Renal Function Groups

The final study group consisted of 38 African American men (*n* = 5) and women (*n* = 33) who were classified as having normal (GFR > 90 mL/min per 1.73 m^2^; *n* = 22) or mildly impaired renal function (GFR 60–89 mL/min per 1.73 m^2^; *n* = 16). The clinical and renal characteristics of normal or mildly impaired renal function groups are presented in Table 1. All clinical and renal characteristics were similar between the two groups with the exception of serum creatinine and GFR. Mean BP levels obtained from office and ABPM measurements are shown in Table 2. The normal renal function group exhibited significantly higher office DBP than the mildly impaired renal function group (*P* < .05). Awake, sleep, and 24-hr SBP, DBP, MAP, and HR were similar between the two groups.

### 3.2. BP Variability

**Figure 1:**
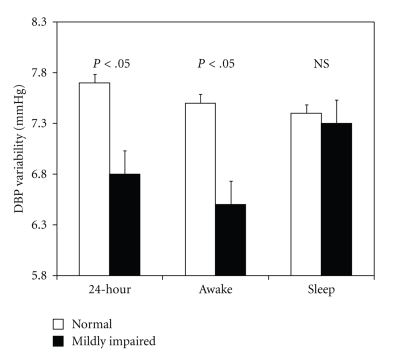
Renal function group differences in DBP variability for awake, sleep, and 24-hour periods.

Figure 1 displays the awake, sleep, and 24-hour diastolic BPV. The mildly impaired renal function group exhibited significantly lower diastolic BPV during the awake period even after adjusting for age, gender, BMI, prior anti-hypertensive medication usage, and mean BP levels from office and ABPM measurements. Similarly, diastolic BPV was also significantly lower in the mildly impaired renal function group during the 24-hour period. This difference was significant after adjusting for gender and BMI, but failed to reach significance after adjusting for age and mean BP levels. There were no statistically significant differences between the two groups for systolic BPV during the awake (normal: 8.2 ± 0.4 versus mildly impaired: 8.4 ± 0.4 mmHg), sleep (normal: 8.7 ± 0.8 versus mildly impaired: 9.2 ± 0.8 mmHg), and 24-hour periods (normal: 8.4 ± 0.4 versus mildly impaired: 8.5 ± 0.4 mmHg). Likewise, MAP variability (MAPV) was similar between the two groups during the awake (normal: 7.5 ± 0.4 versus mildly impaired: 7.0 ± 0.3 mmHg), sleep (normal: 7.7 ± 0.8 versus mildly impaired: 8.6 ± 1.4 mmHg), and 24-hour periods (normal: 7.6 ± 0.4 versus mildly impaired: 7.1 ± 0.2 mmHg).

### 3.3. Rate of BP Variation

**Figure 2:**
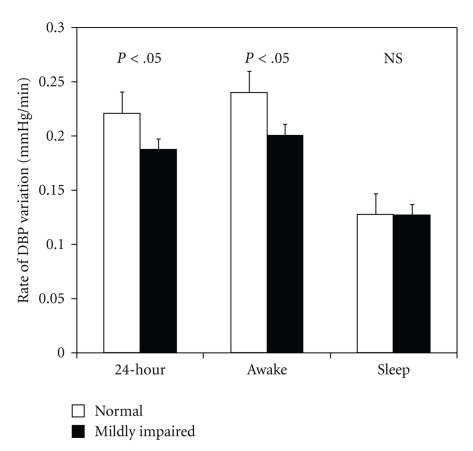
Renal function group differences in rate of DBP variation for awake, sleep, and 24-hour periods.

The rate of variation in DBP during the awake, sleep, and 24-hour periods are presented in Figure 2. The rate of DBP variation was significantly lower in the mildly impaired renal function group during the awake and 24-hour periods. These differences remained significant after adjusting for all clinical characteristics, renal characteristics, and mean BP levels from office and ABPM measurements. There were no statistically significant differences between the two groups for the rate of variation in SBP during the awake (normal: 0.258 ± 0.011 versus mildly impaired: 0.269 ± 0.012 mmHg/min), sleep (normal: 0.146 ± 0.014 versus mildly impaired: 0.158 ± 0.013 mmHg/min), and 24-hour periods (normal: 0.237 ± 0.011 versus mildly impaired: 0.246 ± 0.009 mmHg/min). Similarly, the rate of variation in MAP was not statistically different between the two groups during the awake (normal: 0.237 ± 0.013 versus mildly impaired: 0.227 ± 0.011 mmHg/min), sleep (normal: 0.128 ± 0.016 versus mildly impaired: 0.149 ± 0.024 mmHg/min), and 24-hour periods (normal: 0.218 ± 0.011 versus mildly impaired: 0.208 ± 0.008 mmHg/min).

### 3.4. Regression Analyses

Univariate regression analyses of selected clinical and ABPM variables predicting GFR in the entire study group are presented in Table 3. Diastolic BPV and MAPV during the awake and 24-hour periods, and the rate of variation for DBP and MAP during the awake and 24-hour periods were identified as significant predictors of GFR. Multivariate regression analyses predicting GFR in the entire study group are presented in Table 4. A core regression model was created using variables known to impact renal function: age, BMI, 24-hour SBP, and 24-hour DBP. Each variable identified as a significant predictor of GFR with univariate analyses was then inserted separately into the model. Awake diastolic BPV, 24-hour diastolic BPV, awake rate of DBP variation, and 24-hour rate of DBP variation all remained significant predictors of GFR after adjusting for age, BMI, and mean 24-hour BP. To determine which ABPM parameters were the strongest predictors of GFR, all variables were entered into a forward step-wise linear regression model. In the final model, only the 24-hour rate of DBP variation was included as a predictor of GFR (*β* = .639, *P* < .001), with 40.8% of the variation in GFR accounted for by the final predictor model (*r* ^2^ = .408).

No associations were found for any BPV or BP variation parameter and urinary albumin in the entire study group with univariate regression analyses. When the renal function groups were analyzed separately, urinary albumin was positively associated with the rate of DBP variation in the mildly impaired renal function group during the awake (*β* = .568, *P* < .05) and 24-hour (*β* = .570, *P* < .05) periods. No associations were found for urinary albumin and BPV or BP variation parameters in the normal renal function group.

### 3.5. Plasma and Urinary NOx

Plasma NO*x* (pNO*x*), urinary NO*x* (uNO*x*), and the uNO*x*/pNO*x* ratio were analyzed to investigate the potential role of NO as an underlying mechanism linking renal function to BP fluctuations. pNO*x* levels were significantly higher in the mildly impaired renal function group (normal: 19.2 ± 2.8 versus mildly impaired: 27.2 ± 3.6 *μ*mol/L; *P* < .05). There were no statistically significant differences between the two groups for uNO*x* (normal: 620.3 ± 79.7 versus mildly impaired: 753.5 ± 153.4 *μ*mol/gCr) and the uNO*x*/pNO*x* ratio (normal: 39.5 ± 5.6 versus mildly impaired: 28.4 ± 5.8).

**Figure 3:**
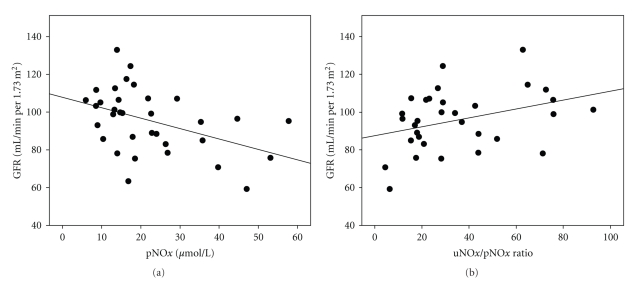
Relationship between GFR and pNO*x* (a) and the uNO*x*/pNO*x* ratio (b).

In univariate regression analyses predicting GFR, both pNO*x* (*β* = −.495, *P* < .05) and the uNO*x*/pNO*x* ratio (*β* = .457, *P* < .01) were significantly associated with GFR (Figure 3).When inserted into a multivariate regression model that included age, BMI, 24-hour SBP, and 24-hour DBP, pNO*x* remained a significant of predictor of GFR (*β* = −.372; *P* < .05). The uNO*x*/pNO*x* ratio also remained a significant predictor of GFR (*β* = −.393; *P* < .05) when inserted into a multivariate regression model that included age and BMI, but did not remain a significant predictor when 24-hour SBP and 24-hour DBP were added to the model. For univariate regression analyses predicting urinary albumin, there was a significant negative association between uNO*x* and urinary albumin (*β* = −.497; *P* < .01) that remained significant when inserted into a multivariate regression that included age, BMI, 24-hour SBP, and 24-hour SBP (*β* = −.625; *P* < .01). 

In order to determine the relationship between a potential BP-buffering mechanism (NO) and fluctuations in BP, univariate regression analyses predicting BPV and BP variation parameters were conducted. A significant negative association existed between pNO*x* and 24-hour diastolic BPV (*β* = −.356, *P* < .05). All remaining renal and ABPM variables showed no significant associations with either pNO*x*, uNO*x*, or the uNO*x*/pNO*x* ratio in the entire study group or in renal function subgroups.

## 4. Discussion

In the present study, we used two indices of BPV to investigate the relationship between renal function and BP variation: the ARV index, which accounts for the order in which BP measurements are obtained, and the time rate of variation, which accounts for the order in which BP measurements are obtained, as well as the time between successive readings. Our results show that normotensive and pre-hypertensive African Americans with mildly impaired renal function exhibit lower diastolic BPV and a lower rate of variation in DBP when compared to African Americans with normal renal function. Moreover, diastolic BPV and the rate of variation in DBP were identified as independent determinants of renal function, as both indices of DBP variation positively correlated with GFR in this cohort of African Americans. 

Our findings conflict with previous studies in diseased populations, as it has been more often shown that greater BP variation is associated with poorer health outcomes in patients with essential hypertension or chronic kidney disease (CKD). Most recently, Manios et al. found that systolic and diastolic BPV and the 24-hour rate of SBP variation were significantly greater in hypertensive patients with a GFR less than 60 mL/min per 1.73 m^2^ when compared to hypertensive patients with a GFR greater than 60 mL/min per 1.73 m^2^ [9]. However, few studies have investigated BP variations in populations without overt clinical disease as the majority of studies investigating BPV have been conducted in hypertensive populations. Given that hypertensive BP loads are directly associated with TOD, and considering that hypertension may be a cause or consequence of baroreceptor and/or endothelial dysfunction; the observed associations between BPV and TOD in these populations could be a result of the effects of a hypertensive BP load on both organs and BP-buffering mechanisms. Thus, our disparate findings may simply be representative of subclinical populations in which some of the BP-buffering mechanisms are still intact, and could suggest that changes in BP regulation mechanisms, resulting in wider BP fluctuations, may occur during the progression of both CKD and hypertension from early, subclincal disease states. This hypothesis is corroborated by our finding that urinary albumin excretion was positively associated with the rate of DBP variation in the mildly impaired renal function group, while no such association was found in the normal renal function group. However, it should be acknowledged that the cross-sectional design of the present study cannot determine whether changes in BP variation promoted CKD or vice versa.

It has been proposed that NO may serve as a BP-buffering mechanism that regulates fluctuations in BP level [10]. Several animal studies have shown that BPV increases with blockade or knockout of endothelial nitric oxide synthase [17, 18], however, to the best of our knowledge, this is the first study to demonstrate a relationship between NO and BPV obtained from ABPM in humans, as a negative association between diastolic BPV and pNO*x* levels was found. Interestingly, the mildly impaired renal function group exhibited greater pNO*x* levels than the normal renal function group. Moreover, when GFR was analyzed as a continuous variable, pNO*x* negatively correlated with GFR and was identified as an independent determinant of renal function. NO plays a major role in renal perfusion and glomerular filtration [19]; therefore, the elevation in pNO*x* with declining GFR observed in the present study may serve as a compensatory mechanism to maintain blood flow to the kidneys in an effort to preserve GFR. This is the first study to compare NO*x* levels in African Americans with normal or mildly impaired renal function; therefore, our findings need to be confirmed; however, in support of NO serving in a compensatory capacity are several animal studies which have also observed a paradoxical increase in NO with declining renal function [20–22]. In keeping with the hypothesis of NO as a BP-buffering mechanism, it therefore seems plausible that the lower BP variation observed in the mildly impaired renal function group may be the result of higher levels of NO production. 

It is interesting to note that higher pNO*x* levels were associated with lower GFR, while no such association was observed between uNO*x* levels and GFR. Because 60%–73% of pNO*x* is excreted renally [23, 24], we also calculated the uNO*x*/pNO*x* ratio in order assess uNO*x* derived from the kidneys after accounting for basal pNO*x* levels. Our findings showed that a lower uNO*x*/pNO*x* ratio was associated with a lower GFR, which could suggest that NO production in the kidney is attenuated with declining renal function. In accordance with these findings, we observed that lower uNO*x* was associated with increased urinary albumin excretion. It has been previously shown that NO production in localized areas of the kidney, including the cortex and outer and inner medulla, is important in the regulation of renal hemodynamics [25]. Moreover, it has been well reported in both clinical and animal studies that deficiencies in NO are associated with kidney damage and/or chronic kidney disease progression [26]. As such, diminished NO production/bioavailability in the kidney could be underlying the finding of reduced BPV in subjects with mildly impaired renal function. 

To the best of our knowledge, this is the first study to investigate BP variations in relationship to renal function in a cohort of African Americans. Therefore, our finding that diastolic BPV and MAPV, but not systolic BPV, were strongly associated with renal function may be the result of underlying race specific contributions to BP regulation. Increased peripheral vascular resistance caused by arterial vasoconstriction is the key determinant of DBP, particularly before 50–60 years of age. In previous studies, when the hemodynamic mechanisms that underlie the BP response were assessed to determine their influence on BP variations from ABPM measurements in different racial groups, systemic vascular resistance was found to play a more prominent role in the regulation of BP in African Africans when compared to whites. In contrast, cardiac output was a prominent contributor to ABPM measurements only in whites and not in African Americans [27]. Moreover, when the hemodynamic responses were compared among racial group after three separate laboratory stressors, the pressor responses in African Americans were found to be mediated to a greater extent by vascular tone when compared to whites [28]. In keeping with the role of peripheral vascular resistance as the key determinant of DBP, it therefore may be reasonable to hypothesize that the strong associations found for the measures of DBP and MAP variations, and not for the SBP variations in the present study, may be attributed to the greater contribution of systemic vascular resistance to BP regulation in African Americans as they are exposed to daily stressors in their natural environment. 

Several limitations of this study must be noted when interpreting these findings. First, our sample size is small. However, because of our extensive exclusion criteria, many confounding variables that may influence renal function were well controlled for. Second, the low frequency of BP measurements with ABPM limits the capacity to optimally assess BP variation. Conversely, the use of ABPM permits the assessment of the variation of BP in a participant's natural environment where they are exposed to the various psychological and environmental stressors which influence their BP on a daily basis. Third, the reproducibility of BP variation may be poor due to the large influence of daily activity on BP variations. Finally, our cross-sectional study design allowed us to assess the relationship between renal function and BPV, but not to assess cause-effect relationships. Prospective studies are needed to determine whether the progression from the early stages of CKD to end-stage renal disease confers changes in the physiological mechanisms that regulate BPV.

In conclusion, our study provides novel information on the relationship between BPV and renal function by assessing their relationship for the first time in a group of normotensive and pre-hypertensive African Americans with normal or mildly impaired renal function. Our findings suggest that both the magnitude and rate of diastolic BPV are reduced in African Americans with mildly impaired renal function, which may in part be the result of a compensatory mechanism in the early stages of hypertension and kidney disease, whereby NO levels in the blood are elevated. These results conflict with previous studies in diseased populations and different races as the relationship between BPV and renal function is reversed in patients with more advanced renal dysfunction and higher BP levels. Future studies should investigate whether changes in BP regulation mechanisms, resulting in wider BP fluctuations, may be involved in the progression of both renal disease and hypertension, and whether these changes could explain the accelerated progression of CKD found in African Americans.

## Figures and Tables

**Table 1:** Clinical and renal characteristics of normal or mildly impaired renal function groups.

	Normal renal function	Mildly impaired renal function
Variable	GFR > 90 mL/min per 1.73 m^2^ (*n* = 22)	GFR 60–89 mL/min per 1.73 m^2^ (*n* = 16)
Clinical characteristics		
^#^Normotensive/pre-hypertensive	7/15 (32%/64%)	7/9 (44%/56%)
^#^Male/female	2/20 (9%/90%)	3/13 (19%/81%)
^#^Med. taper/no Meds	5/17 (23%/77%)	9/7 (56%/46%)
Age (years)	51.0 ± 1.4	54.1 ± 1.5
BMI (kg/m^2^)	33.0 ± 1.0	30.0 ± 1.3
Total fat free mass (kg)	51.8 ± 1.4	51.1 ± 2.7
Total fat mass (kg)	38.9 ± 2.2	33.4 ± 2.9
Total cholesterol (mg/dL)	189.3 ± 6.1	202.6 ± 6.2
LDL cholesterol (mg/dL)	111.1 ± 6.7	119.1 ± 6.5
HDL cholesterol (mg/dL)	61.7 ± 3.8	66.0 ± 4.1
Triglycerides (mg/dL)	82.3 ± 6.6	87.4 ± 8.1
Fasting glucose (mg/dL)	95.2 ± 2.1	94.5 ± 2.4

Renal characteristics		
Urine total volume (mL)	1708.8 ± 160.8	1853.3 ± 267.6
Urinary Na^+^ (mmol/gCr)	93.1 ± 7.6	91.8 ± 10.1
Urinary creatinine (g/24hr)	1.5 ± 0.1	1.5 ± 0.1
Urinary albumin (mg/24hr)	8.9 ± 0.8	8.2 ± 1.0
Serum creatinine (mg/dL)	0.8 ± 0.02	1.0 ± 0.03*
GFR (mL/min per 1.73 m^2^)	105.8 ± 2.1	78.6 ± 2.2*

Values are expressed as means ± SEM. BMI, body mass index; GFR, glomerular filtration rate; HDL, high-density lipoprotein; LDL, low-density lipoprotein.

*Denotes significant difference between groups*; P* < .05.

**Table 2:** Mean BP levels from office and ABPM measurements in normal and mildly impaired renal function groups.

	Normal renal function	Mildly impaired renal function
Variable	GFR > 90 mL/min per 1.73 m^2^ (*n* = 22)	GFR 60–89 mL/min per 1.73 m^2^ (*n* = 16)
Office BP		
SBP (mmHg)	127.3 ± 2.5	122.7 ± 2.8
DBP (mmHg)	81.1 ± 1.7	76.1 ± 2.9*
ABPM		
24-hr SBP (mmHg)	127.1 ± 2.6	126.4 ± 3.0
24-hr DBP (mmHg)	80.3 ± 1.9	76.4 ± 2.3
24-hr MAP (mmHg)	96.5 ± 1.9	93.7 ± 2.3
24-hr HR (beats/min)	75.1 ± 2.4	72.3 ± 2.1
Awake SBP (mmHg)	128.5 ± 2.9	129.0 ± 3.1
Awake DBP (mmHg)	81.7 ± 1.9	78.9 ± 2.5
Awake MAP (mmHg)	97.8 ± 2.0	96.3 ± 2.5
Awake HR (beats/min)	77.2 ± 2.5	73.8 ± 2.3
Sleep SBP (mmHg)	117.0 ± 2.2	116.1 ± 2.5
Sleep DBP (mmHg)	70.3 ± 1.9	66.4 ± 1.9
Sleep MAP (mmHg)	86.7 ± 1.9	83.4 ± 1.8
Sleep HR (beats/min)	67.3 ± 2.7	66.0 ± 2.2
Dip SBP (%)	8.8 ± 1.2	9.8 ± 1.4
Dip DBP (%)	13.8 ± 1.5	15.4 ± 2.3

Values are expressed as means ± SEM. ABPM, ambulatory blood pressure monitoring; BP, blood pressure; DBP, diastolic blood pressure; GFR, glomerular filtration rate; HR, heart rate; MAP, mean arterial pressure; SBP, systolic blood pressure.

*Denotes significant difference between groups; *P* < .05.

**Table 3:** Univariate regression analyses predicting GFR.

Variable	*β*	*P* Value
Age (years)	−0.276	NS
Male Gender	0.005	NS
Med. Taper	−0.282	NS
BMI (kg/m2)	0.179	NS
Fat Free Mass (kg)	0.091	NS
Fat Mass (kg)	0.124	NS
Total Cholesterol (mg/dL)	−0.306	NS
LDL Cholesterol (mg/dL)	−0.152	NS
HDL Cholesterol (mg/dL)	−0.185	NS
Triglycerides (mg/dL)	−0.252	NS
Glucose (mg/dL)	0.068	NS
Awake SBP (mmHg)	−0.038	NS
Sleep SBP (mmHg)	−0.005	NS
24-hr SBP (mmHg)	0.023	NS
Awake DBP (mmHg)	0.131	NS
Sleep DBP (mmHg)	0.154	NS
24-hr DBP (mmHg)	0.213	NS
Awake MAP (mmHg)	0.052	NS
Sleep MAP (mmHg)	0.120	NS
24-hr MAP (mmHg)	0.145	NS
Awake Systolic BPV (mmHg)	0.133	NS
Sleep Systolic BPV (mmHg)	0.169	NS
24-hr Systolic BPV (mmHg)	0.199	NS
Awake Rate of SBP Variation (mmHg/min)	0.114	NS
Sleep Rate of SBP Variation (mmHg/min)	0.135	NS
24-hr Rate of SBP Variation (mmHg/min)	0.153	NS
Awake Diastolic BPV (mmHg)	0.549	<.001
Sleep Diastolic BPV (mmHg)	0.240	NS
24-hr Diastolic BPV (mmHg)	0.524	.001
Awake Rate of DBP Variation (mmHg/min)	0.573	<.001
Sleep Rate of DBP Variation (mmHg/min)	0.228	NS
24-hr Rate of DBP Variation (mmHg/min)	0.626	<.001
Awake MAPV (mmHg)	0.357	.033
Sleep MAPV (mmHg)	0.164	NS
24-hr MAPV (mmHg)	0.431	.008
Awake Rate of MAP Variation (mmHg/min)	0.342	.041
Sleep Rate of MAP Variation (mmHg/min)	0.163	NS
24-hr Rate of MAP Variation (mmHg/min)	0.383	.014

BMI, body mass index; BPV, blood pressure variability; DBP, diastolic blood pressure; GFR, glomerular filtration rate; HDL, high-density lipoprotein; LDL, low-density lipoprotein; MAP, mean arterial pressure, MAPV, mean arterial pressure variability; NS, nonsignificant; SBP, systolic blood pressure.

**Table 4:** Multivariate regression analyses predicting GFR.

Variable	*β*	*P* Value
Awake Diastolic BPV (mmHg)	0.553	.003
24-Hr Diastolic BPV (mmHg)	0.595	.001
Awake Rate of DBP Variation (mmHg/min)	0.538	.002
24-Hr Rate of DBP Variation (mmHg/min)	0.621	.001
Awake MAPV (mmHg)	0.284	NS
24-Hr MAPV (mmHg)	0.308	NS
Awake Rate of MAP Variation (mmHg/min)	0.221	NS
24-Hr Rate of MAP Variation (mmHg/min)	0.259	NS

The listed variables were entered separately into a multivariate regression model containing age, BMI, SBP, and DBP. BPV, blood pressure variability; DBP, diastolic blood pressure; GFR, glomerular filtration rate; MAP, mean arterial pressure; MAPV, mean arterial pressure variability.
